# The clinical significance of premature atrial contractions: How frequent should they become predictive of new‐onset atrial fibrillation

**DOI:** 10.1111/anec.12718

**Published:** 2019-10-11

**Authors:** Eser Durmaz, Baris Ikitimur, Burcak Kilickiran Avci, Adem Atıcı, Ece Yurtseven, Hasan Tokdil, Cansu Ebren, Fuat Polat, Orhan Karaca, Bilgehan Karadag, Zeki Ongen

**Affiliations:** ^1^ Cardiology Department Cerrahpasa School of Medicine Istanbul University‐Cerrahpasa Istanbul Turkey; ^2^ Cardiology Department Goztepe Research and Training Hospital Istabul Turkey; ^3^ Cardiology Department Koc University Hospital Istanbul Turkey

**Keywords:** atrial arrhythmias, clinical, electrophysiology, Holter/event recorders, non‐invasive techniques

## Abstract

**Background:**

Although previous studies reported frequent premature atrial contractions(fPACs) increased the risk of adverse cardiovascular outcomes, especially atrial fibrillation(AF), there is a substantial inconsistency between reports concerning the definition of fPAC. In this study, we aimed to investigate the relationship between fPAC and cardiovascular outcomes, especially AF. We further searched for a cutoff value of fPAC for prediction of AF.

**Methods:**

We retrospectively analyzed the ambulatory 24‐hr Holter monitoring records and 392 patients included. Frequent PAC was defined as more than 720 PAC/24 hr as used for frequent ventricular premature beats. Patients’ baseline characteristics, echocardiographic variables and medical history were recorded.

**Results:**

There were 189 patients with fPAC and 203 patients without fPAC. Patients with fPAC had more comorbidities in terms of hypertension, diabetes mellitus, coronary artery disease and congestive heart failure. CHA2DS2‐VaSc was higher in patients with fPAC. Mean follow‐up duration was 31 months, and the number of patients with new‐onset AF during follow‐up was significantly higher in fPAC group (22% vs. 5%, *p* < .001). fPAC was significantly and independently associated with new‐onset AF and predicted AF with a cutoff value of 3,459 PAC/24 hr, and the risk of AF was 11‐fold higher than those with <3,000 PAC/24 hr. In addition, an increased CHA2DS2‐VaSc score was also associated with new‐onset atrial fibrillation.

**Conclusion:**

In our study, we have demonstrated that fPAC is significantly associated with new‐onset AF, and this association is the strongest among those patients who have more than 3,000 PAC in 24 hr.

## 1 INTRODUCTION

Early depolarization of an atrial tissue leads to premature atrial contraction (PAC). PAC is a frequent finding during routine cardiac evaluation of patients with or without known structural heart disease (Camm, Evans, Ward, & Martin, 1980; Folarin, Fitzsimmons, & Kruyer, 2001). PAC is detected in patients complaining of palpitations very frequently, nearly 100% in the elderly, and 73% in the younger population (Fleg & Kennedy, 1982). Unless PAC causes significant symptoms, it does not necessitate treatment and reassurance is the common daily practice. Although historically the presence of PAC is considered safe, recent data supports its association with future atrial fibrillation (AF), especially when PACs arising from the pulmonary veins are considered (Frost et al., 1995; Haïssaguerre et al., 1998; Kolb et al., 2001). These studies are inconsistent due to the difference in exact definition used for frequent premature atrial contractions (fPAC), and neither Heart Rhythm Society nor European Heart Rhythm Association suggests a cutoff value for frequent PAC.

Since AF is the most frequent sustained arrhythmia and has an adverse impact on cardiac morbidity and mortality, it is important to define patients who are under risk for the development of AF (Kirchhof et al., 2016; Lip & Tse, 2007). Current HRS guidelines define a cutoff value for of premature ventricular contractions (PVC) to become hazardous on left ventricle via negative remodeling and suggest that treatment of PVCs may prevent left ventricular (LV) dysfunction (Al‐Khatib et al., 2018). Likewise, defining a cutoff value for PAC with respect to the risk of future AF may lead to developing a new perspective for management of PACs like management of risk factors, medical therapy and even prophylactic pulmonary vein isolation.

In this study, we aim to investigate the relationship between the frequency of PAC and future atrial fibrillation and to obtain a cutoff value for the prediction of AF. Furthermore, we also investigated the relationship between CHA2DS2‐VASC score and development of AF in patients with frequent PAC.

## 2 MATERIAL/METHOD

The study was conducted in compliance with the Declaration of Helsinki and after permission of Local Ethics Committee of Cerrahpasa School of Medicine. We retrospectively analyzed 24‐hr ECG Holter records of patients referred to our clinic with various indications. Other data including demographic characteristics, echocardiographic parameters, biochemical analyses and drug information were gathered from existing hospital records. The inclusion criteria were as follows: (a) patients over 18 years old, (b) patients successfully followed up after Holter recording, and (c) patients with echocardiographic examination within 3 months of Holter recording. The exclusion criteria were as follows: (a) patients with previously diagnosed sustained arrhythmia including paroxysmal AF, (b) patients with pacemakers, and (c) patients under treatment with a Vaughan Williams class 1 or class 3 antiarrhythmic drug prior to Holter examination. Patients’ prior records and medical treatments were taken into consideration with respect to the previous diagnosis of AF, and in case of doubt, patients were also excluded. Patients’ baseline characteristics were recorded including age, sex, the presence of hypertension (HT), coronary artery disease, heart failure, diabetes mellitus (DM), and previous cerebrovascular accident (CVA) or transient ischemic attack (TIA). Echocardiographic parameters, including left ventricular ejection fraction, pulmonary artery systolic pressure, and left atrial diameter, were also recorded. Clinical outcomes were decided according to hospital records, national record database or through phone call communication; information regarding new‐onset AF, ischemic SVO or TIA and death from any cause were gathered. New‐onset AF was decided with a proof of an ECG or Holter recording of an individual patient. Stroke was decided using national insurance database records, and mortality was decided either using national death declaration database or phone call to whom cannot be reached in the national database systems. CHA2DS2‐VaSc score was calculated as follows: 1 point for the presence of heart failure, hypertension, age between 65 and 74, diabetes mellitus, female gender, and vascular disease, and 2 points for age >74 and previous stroke or TIA.

Since current literature does not suggest a cutoff value for frequent PAC, we considered frequent PAC to be present similar to frequent PVC which was defined as more than 30 PVC in an hour (Al‐Khatib et al., 2018). There were 189 patients with frequent PAC according to this classification (fPAC group), and we also included 203 patients with less than 720 PAC in 24 hr (control group). A total of 392 patients were included in the final analyses.

Holter examination was performed using Cardioline S.P.A. walk 400H ECG Holter system, Cavareno (TN), Italy. Patients whose Holter record duration was less than 22 hr excluded from the study. The majority of patients underwent to 24 hr Holter monitoring but longer monitoring up to 72 hr was performed for patients with a high clinical suspicion of arrhythmia or cardio‐embolic stroke. PAC was defined as follows: an atrial complex with similar QRS morphology to the sinus beat and less than 80% coupling interval to the preceding QRS, when compared to the mean RR interval. Patients with more than three runs of PAC were excluded from the analyses due to indiscrimination of a short‐run paroxysmal atrial fibrillation. Patients with irregularly irregular rhythm and absence of a distinct p wave whether paroxysmal or permanent were considered atrial fibrillation and excluded from the study. When there were more than one Holter records of a particular patient, Holter examination with a higher frequency of PAC was used in the study.

### 2.1 Statistical analyses

All statistical tests were conducted using the Statistical Package for the Social Sciences 19.0 for Windows (SPSS Inc.). The Kolmogorov–Smirnov test was used to analyze the normality of the data. Continuous data were expressed as mean ± *SD*, and categorical data were expressed as percentages. Fisher exact or/and chi‐square test were used to assess differences in categorical variables between groups. The relationships among parameters were assessed using Pearson's or Spearman's correlation analysis according to the normality of the data. Student's *t* test or Mann–Whitney *U* test was used to compare unpaired samples as needed. Survival curves between patient groups were constructed using Kaplan–Meier estimates, and the unadjusted relationship of frequent fPAC with mortality was assessed with the stratified log‐rank test. Univariate and multivariate Cox regression analysis was used to identify independent variables of atrial fibrillation. Independent variables in univariate analysis were CAD, DM, HT, CHF, LVH, LA diameter, CHA2DS2‐VaSc score, and fPAC. After performing univariate analysis for prediction of AF, significantly obtained variables were selected into the multivariate logistic regression analysis with the stepwise method. The results of univariate and multivariate regression analyses were presented as odds ratio with 95% CI. Receiver operating characteristic (ROC) curves were obtained, and the optimal values with the greatest total sensitivity and specificity in the prediction of AF were selected. Significance was assumed at a 2‐sided *p* < .05. The relationship between PAC number and LA diameter was assessed with Spearman correlation analyses.

**Figure 1:**
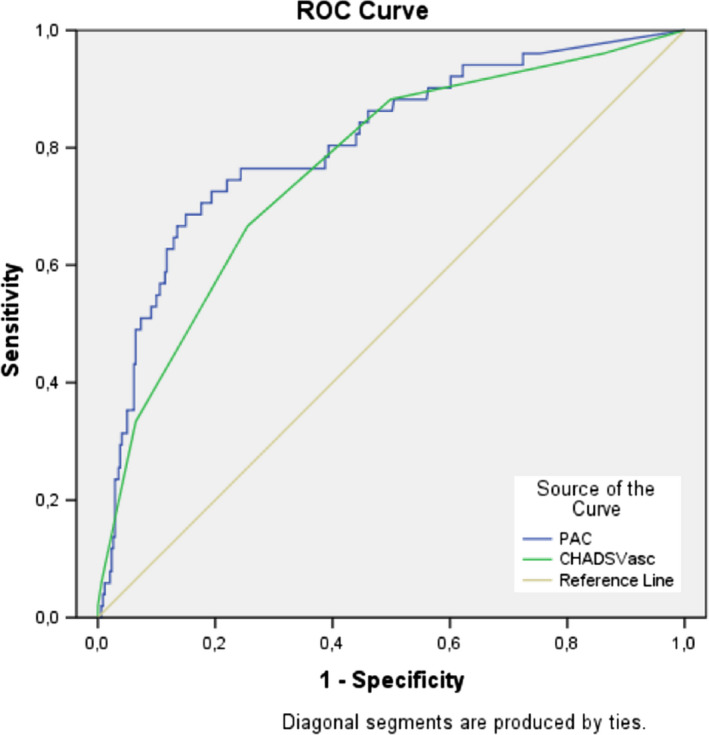
ROC curves of CHA2DS2‐VASC score and fPAC for predicting AF

According to the number of PAC, patients divided into groups of 1,000 and further analyses were made to define a cutoff value. Patients with PAC frequency of more than 8,000 PAC/24 hr placed into the same group. Cox regression analyses were used to determine a cutoff value. There was no significant difference in patients with less than 3,000 PAC, and therefore, further analyses were performed between patients with less than 3,000 and more than 3,000 PAC (Figure 1).

## 3 RESULTS

In total, 392 patients were included in the study. Patients with fPAC were determined to have more comorbidities like hypertension, diabetes mellitus, coronary artery disease, and congestive heart failure (47% vs. 36%, *p* value: .025; 30% vs. 21%, *p* value: .042; 20% vs. 11%, *p* value: .018; 17% vs. 9%, *p* value: .026, respectively) compared to control group (Table 1). Furthermore, structural heart disease in terms of LVH and LA enlargement were more frequent in patients fPAC (48% vs. 26%, *p* value: <.001, for LVH and 39.83 ± 5.85 vs. 36.06 ± 7.46, *p* value: <.001 for LA enlargement). Patients with fPAC had a greater mean CHA2DS2‐VASC score (2.42 ± 1.06 vs. 1.42 ± 0.65, *p*: .001) and significantly higher use of beta‐blocker (39% vs. 27%, *p* value: .011).

**Table 1:** Baseline characteristics of patients with/without fPAC

Patients (*n*)	fPAC (+) (*n*:189)	fPAC (−) (*n*:203)	*p*
Age, years	58.83 ± 14.37	56.34 ± 15.60	.102
Sex (male, *n* [%])	91 (48%)	81 (39%)	.100
HT *n* (%)	90 (47%)	74 (36%)	**.025**
DM *n* (%)	58 (30%)	44 (21%)	**.042**
CVA/TIA *n* (%)	7 (3%)	5 (2%)	.476
CAD *n* (%)	39 (20%)	24 (11%)	**.018**
CHF *n* (%)	32 (17%)	19 (9%)	**.026**
LVH *n* (%)	91 (48%)	54 (26%)	**<.001**
LVEF (%)	54.43 ± 8.23	55.28 ± 9.38	.347
PASP mm Hg	33.67 ± 11.20	32.41 ± 7.58	.193
LAD mm	39.83 ± 5.85	36.06 ± 7.46	**<.001**
CHA2DS2‐VASC score	2.42 ± 1.06	1.42 ± 0.65	**<.001**
PAC number	2,543 (1311–6439)	101 (0–187)	**<.001**
Treatment			
ACEi/ARB	69 (36%)	59 (29%)	.116
Beta‐blocker	74 (39%)	55 (27%)	**.011**
Statins	43 (22%)	36 (17%)	.216

Bold values indicates *p* < .05 are statistically significant.

Abbreviations: ACEi, angiotensin‐converting enzyme inhibitor; ARB, angiotensin receptor blocker; CAD, coronary artery disease; CHF, congestive heart failure; CVA/TIA, cerebrovascular accident/transient ischemic attack; DM, diabetes mellitus; HT, hypertension; LAD, left atrium diameter; LVEF, left ventricular ejection fraction; LVH, left ventricular hypertrophy; PAC, premature atrial contraction; PASP, pulmonary artery systolic pressure.

Mean follow‐up time was 31.06 ± 11.16 months, and there was a significant relationship between fPAC and new‐onset AF: On the other hand, mortality rate and stroke were not different between the groups: mortality 7% vs. 4% (*p*: .196), ischemic stroke 8% vs. 5% (*p*‐value: .149), and atrial fibrillation 22% vs. 5% (*p*‐value < .001), respectively, patients with fPAC vs. without fPAC (Table 2).

**Table 2:** Patients’ outcomes according to presence of fPAC

fPAC	Yes (*n* = 189)	No (*n* = 203)	*p* value
Follow‐up	31.06 ± 11.16	31.10 ± 11.64	.969
Mortality *n* (%)	13 (7)	8 (4)	.196
Stroke *n* (%)	16 (8)	10 (5)	.149
AF *n* (%)	41 (22)	10 (5)	**<.001**

Bold values indicates *p* < .05 are statistically significant.

Abbreviations: AF, atrial fibrillation.

When predictors of atrial fibrillation were analyzed, univariate analyses revealed that coronary artery disease, left atrial diameter, the frequency of premature atrial contractions, and CHA2DS2‐VaSc score to be significantly associated with atrial fibrillation. After adjustment of confounding variables, multivariate analyses revealed coronary artery disease, CHA2DS2‐VASC score and frequency of PAC were independently associated with atrial fibrillation (OR:2.413, 1.292–4.507, CI 95% for coronary artery disease, OR: 1.706, 1.330–2.189, CI 95% for CHA2DS2‐VaSc score; OR: 1.672, 1.321–2.209, CI 95% for fPAC). Further subgroup analysis was performed in order to demonstrate the predictor value of fPAC with respect to LVEF. Although fPAC was significantly associated with new‐onset AF in patients with LVEF < 50 in univariate analysis, multivariate analysis demonstrated no significant relationship between fPAC and new‐onset AF with respect to LVEF. (*p*‐value < .001 in univariate analysis and .859 in multivariate analysis) (Table3).

Further analyses were made to define a cutoff value for prediction of atrial fibrillation among patients with fPAC and patients divided into 8 groups. Patients with <3,000 PAC compared to those over 3,000 PAC did not increase the risk of atrial fibrillation. Cox regression analyses demonstrated PAC frequency >3,459 PAC to increase the likelihood of new‐onset atrial fibrillation 11‐fold. Furthermore, as the frequency of PAC increased, the risk of AF increased proportionally. ROC analyses of fPAC with a cutoff value of 3,459, demonstrated the sensitivity of 83% and specificity of 70% with area under the curve (AUC) 0.80 (95% CI, 0.74–0.87). ROC analyses were made for CHA2DS2‐VaSc score also and when a cutoff value of 2.5 was used it showed a sensitivity of 73%, specificity of 65% and with an AUC of 0.76(95% CI, 0.74–0.87) for prediction of atrial fibrillation (Table 4).

**Table 3:** Univariate and multivariate analyses of predictors of atrial fibrillation

Variable	Univariate			Multivariate		
	OR	95%CI	*p*	OR	95%CI	*p*
Age	0.997	0.973–1.021	.787			
Sex (Male)	0.764	0.414–1412	.391			
HT	1.496	0.769–2.908	.235			
DM	0.756	0.407–1.404	.376			
CAD	2.255	1.160–4.382	**.016**	2.413	1.292–4.507	**.006**
CHF	1.324	0.692–2.535	.396			
LVH	0.600	0.299–1.204	.151			
LAD	1.049	1.002–1.100	**.043**	1.042	0.995–1.092	.080
CHA2DS2‐VASC score	1.830	1.353–2.475	**<.001**	1.706	1.330–2.189	**<.001**
PAC	1.638	1.283–2.092	**<.001**	1.672	1.321–2.209	**<.001**
Beta‐Blocker	1.292	0.690–2.420	.423			

Bold values indicates *p* < .05 are statistically significant.

Abbreviations: CAD, coronary artery disease; CHF, congestive heart failure; DM, diabetes mellitus; HT, hypertension; LAD, left atrium diameter; LVH, left ventricular hypertrophy; PAC, premature atrial contraction.

**Table 4:** ROC analysis of CHA2DS2‐VASC score and fPAC parameters

Parameters	AUC	95% CI	Cutoff value	Sensitivity	Specificity	*p*
CHA2DS2‐VASC score	0.76	0.69–0.83	2.5	73	65	**<.001**
PAC	0.80	0.74–0.87	3,479	83	70	**<.001**

Bold values indicates *p* < .05 are statistically significant.

Abbreviations: AUC, area under receiver operating characteristic curve; CI, confidence interval; PAC, premature atrial contraction.

## 4 DISCUSSION

Principal findings of our study are as follows: (a) frequent PACs are strongly and independently associated with future AF, (b) patients with very frequent PACs, defined as more than 3,000 PAC/24 hr, are under risk of new‐onset AF 11‐fold higher than those without frequent PAC, (c) there is a linear correlation fPAC and risk of AF in patients with very frequent PAC, and (d) patients with high CHA2DS2‐VaSc score are under increased risk of AF compared to those with low CHA2DS2‐VaSc score.

Although there are several studies in the literature which demonstrated a significant relationship between frequent PAC and future adverse cardiovascular outcomes, the exact number of PACs to define the presence of fPAC is not clearly stated and neither HRS nor EHRA have proposed a definition. Since studies which have demonstrated a significant relationship between fPAC and AF have inconsistent definitions for fPAC, we believe a more definitive description of frequent AF is essential for identification of patients under risk of adverse cardiovascular outcomes, especially atrial fibrillation. In this study, we grouped patients as having fPAC when PAC number exceeded 30/hr, the same cutoff value used for HRS to define frequent PVC. In this study, we found that frequent PAC defined as more than 30 PAC per hour was independently associated with AF. Acharya et al. have previously demonstrated more than 100 PAC/day was independently associated with atrial fibrillation, although in this cutoff value was arbitrarily chosen (Acharya et al., 2015). Larsen et al. also used the same cutoff value with our study and they also demonstrated a significant relationship between frequent PAC and AF (Larsen, Kumarathurai, Falkenberg, Nielsen, & Sajadieh, 2015)a. In this study, they concluded that for each 10 PAC per hour increase in PAC frequency, the risk of AF increased by 50%, and however, a definitive cutoff value has not been concluded. Lin et al. have demonstrated that PAC count of more than 76 beats per day has independent predictive value for not only new‐onset AF but also predicts mortality and pacemaker implantation (Lin et al., 2015). We have further analyzed the risk of atrial fibrillation in patients with fPAC and the cutoff value of 3,459 PAC in 24 hr, the risk of atrial fibrillation to be 11‐fold higher than those without fPAC (AUC:0.80, sensitivity: 83% and specificity 70%). Moreover, trend analyses in our study confirmed Larsen et al. In our study, each 1,000 PAC/24 hr increases in the frequency of PAC, increased the risk of AF in a linear fashion. To the best of our knowledge, this is the first study which has defined a cutoff value above which future risk of AF increases dramatically. This is particularly essential for future research in view of considering PAC as a substitute for PVC in the atrium. HRS guidelines about ventricular arrhythmias[9] conclude that PVCs more than 8.000 in 24 hr may cause left ventricular dysfunction via negative remodeling and ablation of this PVC may prevent LV dysfunction. In this line of thought, PAC ablation, especially in patients with more than 3,000 according to our results, may be instrumental in preventing future atrial fibrillation. Prevention of AF is also important as treatment of AF because 35% of ischemic strokes due to AF occurs at first AF attack of a particular patient (Brambatti et al., 2014). The definition of a cutoff value for AF risk is valuable and will open a new horizon for PAC ablation especially those arising from pulmonary veins. Therefore, future research is required to confirm our results.

CHA2DS2‐VaSc score is a validated and guideline‐recommended risk score to estimate thromboembolic risk in patients with nonvalvular AF. In addition, there are several studies which demonstrate predictive value of CHA2DS2‐VASC score besides thromboembolism in AF, such as prediction of left atrial dysfunction or prediction of thromboembolism in patients with sinus rhythm (Mitchell et al., 2014; Saha et al., 2011). Individual parameters of CHA2DS2‐VaSc score are also the risk factors for occurrence of AF such as age, hypertension, coronary artery disease, and diabetes mellitus. Therefore, it is logical to assume that increased CHA2DS2‐VaSc score could increase the risk of AF. Previous studies demonstrated that in patients with frequent AF, CHA2DS2‐VaSc score increased the risk of atrial fibrillation which are comparable to our results (Chong et al., 2012; Larsen et al., 2015; Suzuki et al., 2013). In our study, we also found an independent association between CHA2DS2‐VaSc score and AF in patients with fPAC. Shinya et al. have demonstrated the same relationship with AF and CHADS_2_ score in patients with fPAC (Suzuki et al., 2013). We have additionally investigated a cutoff value for CHA2DS2‐VASC score for prediction of new‐onset AF; ROC analyses demonstrated that a cutoff value of 2.5, CHA2DS2‐VaSc score had the sensitivity of 73% and specificity of 65%, and AUC was 0.76 for prediction of new‐onset AF.

There are inconsistent reports in the literature about the association between fPAC and ischemic stroke. Larsen et al demonstrated a significant relationship between fPAC and ischemic stroke whereas Shinya et al failed to demonstrate such a relationship. In our study, we did not find a significant relationship between fPAC and stroke. In a meta‐analysis, Huang et al concluded that further research needed to clarify this relationship (Huang et al., 2017). In our study, there was no significant relationship between stroke and fPAC. Considering risk factors for ischemic stroke, atrial fibrillation, and fPAC, there are several common risk factors such as hypertension, advanced age, or diabetes mellitus. Therefore, it is logical to assume fPAC and ischemic stroke are related, but current literature does not have enough evidence to support the independent value of fPAC for prediction of stroke. Furthermore, considering the low annual incidence of ischemic stroke, our study and previous studies included a relatively small number of patients and a limited follow‐up period; therefore, the observed inconsistency between these studies is not surprising. In this regard, for a clarified result, studies with larger patient populations and longer follow‐up time are needed for assessment of stroke‐fPAC relationship.

In our study, patients with fPAC have more comorbidities in terms of HT, DM, CAD, and CHF and more structural abnormalities such as left ventricular hypertrophy which were previously demonstrated to alter the function of left atrium and considered as risk factors for development of atrial fibrillation (Buggey & Hoit, 2018; Mondillo et al., 2011). Kuppahally et al. have demonstrated the dysfunction of the left atrium is associated with atrial fibrillation (Kuppahally et al., 2010). Therefore, fPAC may be a consequence of diseased left atrium rather than a risk factor by itself. Moreover, as AF cause left atrial dysfunction and trigger atrial fibrillation, as known as AF begets AF, fPAC may cause left atrial dysfunction and trigger AF. Further research is needed in this regard.

### 4.1 Limitations

There are several limitations of our study. First, the majority of patients in our study underwent 24 hr of Holter monitoring which occasionally cannot detect patients with paroxysmal AF. However, in our daily practice, our clinical approach is 72 hr of Holter monitoring in high‐risk patients such as patients presenting with cardio‐embolic stroke or high suspicion of arrhythmia. Furthermore, although relatively long enough follow‐up duration of our study, this duration may not be enough to elucidate adverse events, especially ischemic stroke which has low annual incidence. The other limitation of our study is lower number of patients with high CHA2DS2‐VaSc score which may have substantial impact on outcomes such as new‐onset AF and stroke. Further studies with higher number of patients which have more balanced distribution and longer follow‐up are required to validate our results.

## 5 CONCLUSION

Our results indicate that patients with frequent PAC are under risk of new‐onset atrial fibrillation, especially those with frequency of more than 3,000 PAC/24 hr. In addition, patients with high CHA2DS2‐VASC score are under increased risk of future AF. Further studies are needed to confirm our cutoff value which may have a role for predicting development of AF.
